# Self-reported narcissistic traits in patients with addiction through the lens of the ICD-11 model for personality disorders

**DOI:** 10.3389/fpsyt.2022.1041480

**Published:** 2022-11-25

**Authors:** Karel D. Riegel, Lucia Schlosserova, Tadeas S. Zbornik

**Affiliations:** Department of Addictology, First Faculty of Medicine, Charles University and General University Hospital in Prague, Prague, Czechia

**Keywords:** ICD-11, DSM-5 AMPD, narcissistic personality disorder, dissociality, personality functioning, addiction

## Abstract

**Background:**

There is a presumption that pathological narcissism, or narcissistic personality disorder *per se*, can be considered a precursor to addiction. Although the ICD-11 model does not distinguish specific personality disorders, narcissistic psychopathology should be captured through personality trait qualifiers.

**Objectives:**

To verify the capacity of the ICD-11 model in the detection of narcissistic psychopathology in patients with addiction; to test its discrimination capacity, convergent validity, and specificity toward the gender and the type of addiction.

**Materials and methods:**

Two samples were employed in the study. Sample 1 (*n* = 421) consisted of patients with addiction; Sample 2 (*n* = 567) consisted of general population volunteers. Age range was 18–75 years and a battery of self-assessment questionnaires containing Personality Inventory for DSM-5–Brief Form Plus Modified; Triarchic Psychopathy Measure; Hypersensitive Narcissism Scale; and Level of Personality Functioning Scale-Self-Report was administered by pencil-and-paper method.

**Results:**

The following was confirmed: (1) capacity of the ICD-11 model in relation to capture narcissistic pathology; (2) the differentiation capacity between the clinical and non-clinical population; (3) gender specificity in relation to grandiose and vulnerable narcissism; (4) the connection between the overall degree of impairment in personality functioning and most of trait qualifiers; (5) certain specifics of patients with addiction in relation to the type of addiction.

**Conclusion:**

Results support the empirical and clinical relevance of the ICD-11 model in capturing narcissistic pathology in addicted patients. Clinical implications concerning assessment and treatment in addiction settings, and certain limits regarding the Anankastia domain are discussed.

## Introduction

The search for connections between pathological narcissism and addictions has a long tradition, especially among psychoanalytically oriented authors. Wurmser (1), for example, with reference to Kohut’s self-psychology, postulated the basic psychological conditions leading to addiction. These conditions include, in addition to psychosocial factors and specific triggering conditions: (1) latent narcissistic personality disorder (NPD) with internal contradictions between idealized, grandiose ideas and the experience of oneself and others (narcissistic conflict); (2) emotional disturbance, accompanied by disillusionment, anger, a feeling of emptiness or severe anxiety (narcissistic crisis). This challenging condition motivates a person to search for a drug that should alleviate it.

The variable prevalence rates of co-morbid NPD and substance use disorder (SUD)^1^ in different settings (2, 3) imply a functional range in people given this diagnosis. On the other hand, the method of establishing the NPD diagnosis itself can significantly distort information about the occurrence of manifestations of pathological narcissism in the addicted population. To date, several comprehensive reviews of pathological narcissism have been published [e.g., (4–7)], which document the changing stages and issues leading to difficulties in integrating scientific and clinical knowledge of narcissistic disorder. Narcissism is inconsistently defined and assessed across clinical psychology and psychiatry thus, what is described, or empirically measured, is often difficult to synthesize with each other. With an attempt to synthesize theories of narcissism with research findings, Pincus and Lukowitsky (8) proposed that pathological narcissism is best conceptualized by a hierarchical model. In their view, pathological narcissism is essentially characterized by a combination of three psychodynamic phenomena: dysfunctional self-regulation, dysfunctional emotion regulation, and dysfunctional interpersonal relationships, which they consider to be the basis of pathological narcissism and place it on a continuum between the two basic prototypes: at one end of the spectrum is the prototype of grandiose (arrogant, indifferent) narcissism and at the other end is the prototype of vulnerable (hypervigilant, exhausted) narcissism. Authors state that both grandiose and vulnerable narcissism can manifest both covertly and overtly.

Regarding a common etiology between dissociality/psychopathy and substance abuse built upon tendencies to novelty-seeking, impulsivity, and disinhibition (9–11), a third prototype which includes narcissistic people within the range of malignant narcissism, antisocial/dissocial personality disorder, and psychopathy should be mentioned. The characteristics of manipulativeness, exploitativeness, and callousness can be considered general antagonistic personality traits in this group of narcissistic personalities, most closely shared between psychopathy and various narcissistic dimensions [e.g., (12, 13)]. This “classical” conceptualization of psychopathy with narcissistic traits related to self-centered antagonism takes on special importance also in connection with the conceptualization of NPD and pathological narcissism in the upcoming ICD-11 model for personality disorders and the DSM-5 Alternative Model of Personality Disorders (AMPD). However, recent research with the Trifurcated Model of Narcissism (14) has demonstrated that, from the perspective of current empirical conceptualizations, some well-established psychopathy assessment instruments [e.g., the Triarchic Psychopathy Measure (TriPM)] may not adequately capture narcissistic traits relative to agentic extraversion, i.e., the second major component related to grandiose narcissism (alongside self-centered antagonism) (15). On the other hand, another study (16) in relation to the TriPM demonstrated a significant overlap of its Boldness domain with the domains of low Neuroticism and high Extraversion according to the general and pathological five-factor model of psychopathology, which is adopted in AMPD and to a large extent also in the ICD-11 model of maladaptive personality traits.

Although both the ICD-11 classification of personality disorders and the AMPD work with the overall impairment in self/other functioning and trait domains qualifiers that contribute to the individual expression of personality disorder (i.e., Negative Affectivity, Detachment, Disinhibition, Dissociality/Antagonism, Anankastia, and Psychoticism^2^) (17), the two models differ significantly in the way they operationalize narcissistic psychopathology. While hybrid AMPD combines a dimensional approach with the maintenance of a categorical diagnosis of NPD (18), the ICD-11 model remains purely dimensional (19). Even though, from the perspective of personality traits, NPD determined in AMPD based on grandiosity and attention seeking (i.e., both facets of the Antagonism domain) may be advantageous for the clinician in terms of more straightforward decision making, the diagnosis is relying rather on overt and one-sided determinants of grandiosity while the vulnerable features are not sufficiently captured (20, 21). The emphasis on self-centeredness as an essential feature of narcissistic psychopathology is also preserved in the ICD-11 model, which considers Dissociality as a core trait domain, overarching all forms of pathological narcissism (22). As the Dissociality trait domain qualifier may not appear very specific for narcissism because it would also characterize dissocial PD and psychopathy, according to Bach et al. (23) it seems reasonable to combine Dissociality with Anankastia domain when describing the grandiose narcissism prototype and with Negative Affectivity domain when describing the vulnerable narcissism prototype.

If we consider the latter distinction based on how an individual perceives and processes external feedback, some parallel might be found with the reward sensitivity pathway hypothesis predisposing NPD patients to substance abuse based on dopaminergic/opioidergic hyperreactivity (24, 25). Moreover, Salazar et al. (26) in relation to the dopaminergic effect in SUD patients, have recently proposed lack of empathy and perceptions of invulnerability (i.e., common features of self/other maladaptive functioning in SUDs) as mechanisms of self-esteem regulation by means of self-inducing a “narcissistic state.” From this perspective some individuals with SUDs might engage in taking drugs to boost their inherent feeling of superiority over others. Thus, the general assumption about the low self-esteem of SUD patients becomes questionable. Taken together, all these findings support the need for thorough evaluation of narcissistic traits and their interpretation in the context of overall personality functioning when the issue of NPD and pathological narcissism in SUDs is in question. Moreover, there is evidence that knowledge about the patient’s personality might help to improve the working alliance and prevent premature dropout or relapse, more frequent in SUD patients with comorbid Cluster B PDs (27–29).

Considering extensive comorbidity within PD types based on the traditional categorical approach (30), as well as the recommendation of the DSM-5 to separate narcissistic traits from symptoms that may develop in connection with the continued use of addictive substances (18), there is a need for diagnostic tools that allow a comprehensive and reliable assessment of the manifestations of pathological narcissism in addiction clients at different stages of the treatment process. It was proved that a common factor underlying PDs accounts for much of the comorbidity between Axis II personality disorders and substance dependence, whereas residual Cluster B factor was more highly related to the substance dependence diagnoses than was the General PD factor (31). These findings to some extent correspond with the results of Creswell et al. (32), who proved the incremental validity of the AMPD Antagonism and Disinhibition domains as assessed by the Personality Inventory for DSM-5 (PID-5) (33) in predicting alcohol problems above and beyond general personality dysfunction. On the other hand, Papamalis (34) recently highlighted the role of general personality dysfunction by pointing to certain adaptive mechanisms of personality functioning such as self-control, social concordance, and stable self-image as key predictors of addiction treatment completion. In addition, low level of the social concordance domain indicating “the ability to value someone’s identity, withhold aggressive impulses toward others and work together with others” remained one of the most significant predictors of dropout. As these antagonistic features associated with low agreeableness generally describe disagreeable, distrustful, suspicious, oppositional, manipulative, and/or arrogant individuals, who often lack insight into the maladaptivity of their traits (35) a direct connection with manifestations of pathological narcissism can be considered.

The issue of gender differences is also a current topic of pathological narcissism research. Green et al. (36) in a recent review pointed out the shortcomings of the hitherto prevailing way of evaluating narcissistic pathology, which primarily focuses on grandiose aspects that closely resemble prevailing masculine norms. A gender imbalanced approach to the assessment of narcissistic psychopathology may thus lead to the fact that manifestations of pathological narcissism in women are underdiagnosed, which can be manifested by shyness, shame, hypersensitivity, and low self-esteem, i.e., components typical of the vulnerable narcissistic prototype, rather than the aggressive self-assertion typical of the grandiose prototype (5).

Despite the obvious evidence of the link between pathological narcissism and SUDs, as well as evidence of the clinical utility and validity of NPD (37), a limited number of studies can be found in the current literature that deal with the issue of narcissism in patients with addictions from the perspective of dimensional models for PDs. This study seeks to fill a research gap regarding the extent to which self-reported personality pathology in addicted patients can be attributed to traits of pathological narcissism or NPD. Due to the close overlap between the ICD-11 PD model and AMPD, algorithms to evaluate the five trait domains of the proposed ICD-11 based on PID-5 have been recently proposed (38, 39). Additionally, newly developed modified version of the Personality Inventory for DSM-5–Brief Form Plus (PID5BF+M) (40), which has demonstrated satisfactory psychometric properties both as an instrument extracted from the 220-item version of the PID-5 and as a stand-alone measure (41), allows for the parallel assessment of maladaptive traits across both models. Similarly, from the perspective of the overall personality functioning, the Level of Personality Functioning Scale-Self-Report (LPFS-SR) (42) proved its validity in assessing general personality severity within the ICD-11 model above and beyond AMPD for which it was primarily designed (43, 44).

Based on the previous knowledge about the connection between narcissistic psychopathology and SUDs in relation to general personality dysfunction and dimensional personality traits (e.g., 31, 32, 33, 34), the main goal of the present study was to clarify to what extent the ICD-11 model for PDs affects narcissistic phenomena in relation to both prototypes of pathological narcissism, i.e., grandiose, and vulnerable. In this regard, we hypothesized that the general personality functioning impairment based on the LPFS-SR would cover most manifestations of pathological narcissism. In relation to the domains of personality traits based on the PID5BF+M, we assumed the confirmation of the relation of the domains Dissociality and Anankastia to the grandiose type of narcissism and the domains of Dissociality and Negative Affectivity in relation to the vulnerable type of narcissism, as postulated by Bach et al. (23). Moreover, it is hoped that findings will enlarge the current state of knowledge about the application of the new model in terms of discrimination capacity, convergent validity, and its specificity toward the gender and the type of addiction. These findings might potentially contribute to the further development of the ICD-11 model and its clinical usability in this specific clientele.

## Materials and methods

### Samples and procedures

Two samples were used in this study: a clinical sample of SUD patients (Sample 1) and a control sample of general population volunteers (Sample 2). Age over 18 and the ability to read fluently in the Czech language were the inclusion criteria in both samples; the diagnosis of addiction was the inclusion criterion in the clinical sample; the exclusion criteria in the clinical sample were the presence of acute psychotic symptoms, current intoxication or organic brain disorders accompanied by relevant cognitive impairment. Participation in the study was voluntary and anonymous for all respondents, and all participants were asked to give their informed consent to participate in the study, which they had the opportunity to withdraw at any time without stating the reason. Participants were not rewarded for their participation in the study; however, if they were interested in feedback, they could provide us with their email address, which was held apart from anonymously analyzed data. Trained administrators conducted the data collection. The Ethics Committee of the General University Hospital in Prague approved the study protocol and the informed consent form. The total number of (*n* = 988) respondents was included in the study, with certain specifics in relation to the analyses performed (see Plan of analysis for details).

Sample 1 (*n* = 421) consisted of patients with addiction from seven facilities providing the specialized higher threshold addiction treatment across the Czechia. To cover the widest possible spectrum, institutions providing outpatient and inpatient hospital care, as well as therapeutic communities, were approached for cooperation. On the other hand, we excluded lower threshold services, such as harm reduction interventions and opioid substitution programs or detoxification units, regarding the risk of data distortion due to acute intoxication or low willingness of patients to cooperate. The diagnosis of addiction was based on a standard clinical interview according to the ICD-10/DSM-IV criteria, conducted by experienced psychiatrists. Additional information about the individual history of substance abuse or gambling were extracted from the demographic questionnaire. The representation of respondents by type of addiction was as follows: alcohol (*n* = 314, 74.6%); illicit drugs (*n* = 235, 55.8%); prescribed medication (*n* = 138, 32.8%); gambling (*n* = 64, 15.2%).^3^ The number of participants according to gender was disproportionate: males (*n* = 290, 68.9%) prevailed over females (*n* = 129, 30.6%), two respondents did not indicate their gender (0.5%). Age range was 18–73 years (*M* = 39.3, SD = 10.9). The distribution of the highest attained education was as follows: primary (elementary school) education 24.8%; secondary (high school) education 62.7%; and university education 12.5%. The patients were enrolled in the study in collaboration with the Department of Addictology (General University Hospital in Prague), psychiatric hospitals in Červený Dvůr, Dobřany, Prague-Bohnice, Havlíčkův Brod, and Horní Beřkovice, and five therapeutic communities covered by the psychiatric hospital in Bílá Voda.

Sample 2 (*n* = 567) consisted of university students from various fields of study, working volunteers and pensioners. Recruitment was carried out using convenience sampling. Additional information about the individual history of substance abuse or gambling were extracted from the demographic questionnaire. The representation of respondents by type of addiction was as follows: alcohol (*n* = 45, 6.7%); illicit drugs (*n* = 52, 7.7%); prescribed medication (*n* = 32, 4.7%); gambling (*n* = 2, 0.3%). The number of participants according to gender was again disproportionate: females (*n* = 370, 65.2%) prevailed over males (*n* = 192, 33.9%) five respondents did not indicate their gender (0.9%). Age range was 18–75 years (*M* = 32.9, SD = 12.2). The distribution of the highest attained education was as follows: primary (elementary school) education 11.9%; secondary (high school) education 49.0%; and university education 39.1%.

In the Sample 1, the data collection was conducted either individually (in case of outpatients) or in a group by the paper-and-pencil method during several appointments in the aforementioned institutions. Suitable respondents were included in the study following consultations with the heads of the psychiatric departments, who also obtained their informed consent and allocated a time slot in the daily program for the administration of the test battery. The attending clinician introduced the administrators to the respondents. In the Sample 2, volunteers were asked to complete the test individually using the paper-and-pencil method. All respondents were asked to carefully read the instructions before starting the test, while the administrator was present to assist with technical queries.

### Instruments

#### Personality Inventory for DSM-5–Brief Form Plus Modified (PID5BF+M)

We used the self-report PID5BF+M to operationalize the ICD-11 and DSM-5 domains of personality traits. The six domains have been calculated based on the average scores of the three primary facets of each particular domain: Negative Affectivity has been calculated from the average scores of the facets of emotional lability, anxiousness, separation insecurity; Detachment from the facets of withdrawal, anhedonia, intimacy avoidance; Dissociality/Antagonism from the facets of manipulativeness, deceitfulness, grandiosity; Disinhibition from the facets of irresponsibility, impulsivity, distractibility; Psychoticism from the facets of unusual beliefs and experiences, eccentricity, perceptual dysregulation; and Anankastia from the facets of perfectionism, rigidity, and orderliness. The operationalization of the Anankastia domain in the PID5BF+M is based on the extraction of the three subfacets of orderliness, rigidity, and perfectionism from the PID-5 composite facet rigid perfectionism, which corresponds to the originally proposed 37-facet version of PID-5 (45). At the same time, PID5BF+M omits the perseveration facet as the primary facet of the Anankastia domain because perseveration was originally intended to capture signs of Negative Affectivity (46). The complete PID5BF+M thus consists of 18 facets assessed through 36 items (two items per facet), rated on a four-point Likert scale ranging from 0 (“very untrue or often untrue”) to 3 (“very true or often true”). For the purposes of this study, we employed the PID5BF+M extracted from the 220-item version of PID-5, in accordance with previous studies (40, 47). In the present study, the Cronbach’s alphas for 6 PID5BF+M domains ranged from 0.61 (Anankastia) to 0.77 (Dissociality/Antagonism), indicating acceptable internal consistency given the small number of comprising items.

#### Level of Personality Functioning Scale-Self-Report (LPFS-SR)

To assess the overall level of impairment in personality functioning we administered LPFS-SR, a comprehensive self-report measure for assessing criterion A of the AMPD. Due to the apparent similarity of the two models, the LPFS-SR can also be used to assess a general PD severity according to ICD-11 criteria (43, 44). It features descriptions of five different levels of impairment in the domains of Identity, Self-Direction, Empathy, and Intimacy. It includes 80 items that are rated on four-point Likert scale ranging from 1 (“totally false, not at all true”) to 4 (“very true”). In the present study, the Cronbach’s alpha for the LPFS-SR total score was 0.95. The Cronbach’s alphas for 4 LPFS-SR domains ranged from 0.89 (Intimacy) to 0.98 (Identity), indicating excellent internal consistency.

#### Triarchic Psychopathy Measure (TriPM)

TriPM is a 58-item self-report instrument consisting of three scales: Meanness, Boldness, and Disinhibition. The measure uses a four-point Likert scale ranging from T (“True”) to F (“False”), requiring the respondents to rate the degree to which each item applies to them. The tool was developed by Patrick et al. (48), studies found evidence for very good internal consistencies, good test–retest reliability, and strong validity consistent with the triarchic model of psychopathy (49). For purposes of the present study, TriPM was used as a predictor of grandiose narcissism prototype. Although, unlike other instruments (e.g., Pathological Narcissism Inventory), it is not primarily a method designed to assess grandiose narcissism, we preferred TriPM due to its availability in Czech. The Cronbach’s alpha for the TriPM total score was 0.87. The Cronbach’s alphas for three TriPM domains ranged from 0.84 (Meanness) to 0.87 (Boldness) indicating excellent internal consistency.

#### Hypersensitive narcissism scale (HSNS)

HSNS is a 10-item self-report scale measuring a covert aspect of narcissism (50). Each statement should be evaluated on the five-point Likert scale ranging from 1 (“very uncharacteristic or untrue, strongly disagree”) to 5 (“very characteristic or true, strongly agree”). For purposes of the present study, HSNS was used as a predictor of vulnerable narcissism prototype. Like in the case of the TriPM above, we preferred HSNS to assess vulnerable narcissism due to its availability in Czech. Its Cronbach’s alpha was 0.70, indicating good internal consistency.

#### Sociodemographic questionnaire

In addition to all above-mentioned measures, basic demographic data (age, gender, educational status, and type of addiction) were collected by means of a self-designed questionnaire.

### Plan of analysis

To minimize dropouts, we evaluated each instrument separately as part of data cleaning procedure. This means that, e.g., an invalid protocol due to missing items in one of the methods did not necessarily mean that the respondent was excluded from the study, if his/her answers in the other methods allowed for a valid evaluation based on the instrument manual. Moreover, we used the PID-5 Response Inconsistency Scale (PID-5-RIS) developed by Keeley et al. (51), which has proven successful in detecting random responses in the original version of PID-5 and has been verified by several recent studies (52–54). In line with these studies, we excluded respondents with a PID-5-RIS score ≥17.

The internal consistency of each scale in both samples was estimated using Cronbach’s alpha. The majority of Cronbach’s alphas were ≥ 0.70 (with three exceptions in relation to the PID5BF+M Anankastia, Psychoticism, and Detachment domains), so the data were considered mostly internally consistent. In addition, for measurement invariance testing between PID-5 and PID5BF+M, we used a chi-square difference test, as well as relative differences in additional fit indices. For absolute model fit, the cut-off values of CFI > 0.90 and RMSEA < 0.08 were considered, as well as 90% confidence intervals of RMSEA.

As the data did not meet the conditions for the regression model and the errors have a normal distribution, general linear models using Ordinary Least-Squares regression were applied to show the effect of TriPM and HSNS on LPFS-SR and PID5BF+M capacity to capture narcissistic psychopathology first in Sample 1 and subsequently in Sample 2.

The Shapiro–Wilk test was used to test the data distribution. As the data were predominantly not normally distributed, non-parametric tests were used. Scales TriPM, HSNS, LPFS-SR, as well as PID5BF+M domains of Negative Affectivity, Detachment, Dissociality/Antagonism, Disinhibition, Psychoticism, and Anankastia were compared between Sample 1 and Sample 2 and between gender by Mann–Whitney *U* test. The addiction type specificity in Sample 1 toward the scales was tested by Kruskal–Wallis *H* test. All *p*-values were corrected for multiple testing by the Bonferroni adjustment.

All tests and models were done in IBM SPSS Statistics 25 (55).

## Results

### Measurement invariance

To test the invariance between the original 220-item PID-5 and modified PID5BF+, we estimated a five-domain AMPD trait model in accordance with the five domains qualifiers defined in ICD-11. Confirmatory factor analysis confirmed excellent fit with the original, 220-item PID-5, χ^2^_(120)_ = 532.5, *p* < 0.001, CFI = 0.921, RMSEA = 0.061, 90% RMSEA CI [0.056, 0.066].

### Capacity to capture narcissistic pathology

In the predictive part of our analyses, we employed TriPM domains of Boldness, Disinhibition, and Meanness, as well as HSNS total score as predictors of the narcissistic features latently contained in LPFS-SR and domains of PID5BF+M. As can be seen from Table 1, we obtained quite similar results for both samples. In both samples, the largest proportion (almost 50%) of variability based on HSNS and TriPM can be significantly explained in LPFS-SR. In contrast, the variability of the Anankastia domain in both samples cannot be significantly explained by either HSNS or TriPM. In the case of the Negative Affectivity domain, approximately 40% of the variability can be explained in both samples by HSNS and TriPM, however, the TriPM Meanness domain proved to be a significant negative predictor only in Sample 2. Similarly, in the case of the Detachment domain, while in Sample 2 the TriPM Disinhibition domain emerged as a significant predictor, in Sample 1 this relationship was not demonstrated. The predictive capacity of the TriPM Disinhibition domain also proved weak in the case of the Dissociality/Antagonism domain, but only in Sample 1. Nevertheless, almost 40% of variability of the Dissociality/Antagonism domain in Sample 1 can be still explained by HSNS and TriPM. The TriPM Boldness domain emerged as a statistically significant negative predictor of the Disinhibition domain, but only in Sample 1, while the TriPM Meanness domain emerged as a weak but significant predictor of the Psychoticism domain only in Sample 2.

**TABLE 1:** Prediction of narcissistic features in LPFS-SR and PID5BF+M domains in both samples.

S1		(Intercept)	HSNS total	TriPM_BOL	TriPM_DIS	TriPM_MEAN	R-squared
LPFS-SR	Estimate	283.312***	4.546***	−1.673***	1.575***	1.202***	0.451
	SE	2.476	0.474	0.289	0.253	0.323	
NEF	Estimate	1.231***	0.034***	−0.025***	0.019***	–0.002	0.398
	SE	0.012	0.006	0.003	0.003	0.004	
DET	Estimate	0.921***	0.018***	−0.024***	–0.001	0.017***	0.224
	SE	0.028	0.005	0.003	0.003	0.004	
DISO/ANT	Estimate	0.795***	0.028***	0.015***	0.007*	0.025***	0.38
	SE	0.028	0.005	0.003	0.003	0.004	
DIS	Estimate	1.222***	0.018***	−0.011***	0.031***	–0.002	0.434
	SE	0.027	0.005	0.003	0.003	0.004	
PSY	Estimate	0.855***	0.028***	0.004	0.019***	0.002	0.291
	SE	0.028	0.005	0.003	0.003	0.004	
ANA	Estimate	1.053***	–0.006	0	0	0.005	0.008
	SE	0.033	0.006	0.004	0.003	0.004	

**S2**		**(Intercept)**	**HSNS total**	**TriPM_BOL**	**TriPM_DIS**	**TriPM_MEAN**	**R-squared**

LPFS-SR	Estimate	215.715***	2.741***	−1.632***	3.175***	1.035***	0.486
	SE	1.665	0.362	0.229	0.329	0.31	
NEF	Estimate	0.772***	0.038***	−0.014***	0.028***	−0.014***	0.391
	SE	0.021	0.005	0.003	0.004	0.004	
DET	Estimate	0.521***	0.015***	−0.013***	0.011**	0.015***	0.255
	SE	0.018	0.004	0.002	0.004	0.003	
DISO/ANT	Estimate	0.463***	0.015***	0.011***	0.016***	0.022***	0.343
	SE	0.017	0.004	0.002	0.003	0.003	
DIS	Estimate	0.651***	0.016***	–0.004	0.035***	0	0.364
	SE	0.017	0.004	0.002	0.003	0.003	
PSY	Estimate	0.448***	0.018***	0.004	0.021***	0.007*	0.26
	SE	0.017	0.004	0.002	0.003	0.003	
ANA	Estimate	1.161***	0.002	–0.001	0.002	–0.003	0.003
	SE	0.025	0.005	0.003	0.005	0.005	

S1 and S2, Sample 1 and Sample 2; NEF, Negative Affectivity; DET, Detachment; DISO/ANT, Dissociality/Antagonism; DIS, Disinhibition; PSY, Psychoticism; ANA, Anankastia; TriPM_BOL, Boldness; TriPM_DIS, Disinhibition; TriPM_MEAN, Meanness. *p < 0.05; **p < 0.01; ***p < 0.001.

### Discrimination capacity

As can be seen from Table 2, it was found that respondents from Sample 1 scored significantly higher in all observed scales at *p* < 0.001, only in Anankastia at *p* < 0.05. From the perspective of gender, in Sample 1, differences were found between TriPM (*U* = 12968.5; *p* < 0.001) where men scored significantly higher, HSNS (*U* = 15685.5; *p* < 0.013) where women scored significantly higher, and PID5BF+M domain Negative Affectivity (*U* = 9439.0; *p* < 0.001) where women scored significantly higher. In Sample 2, significant differences were found between TriPM (*U* = 23853.5; *p* < 0.001) where men scored significantly higher, HSNS (*U* = 29858.0; *p* < 0.012) where women scored significantly higher, and PID5BF+M domains Negative Affectivity (*U* = 20090.5; *p* < 0.001) where women scored significantly higher, and Dissociality/Antagonism (*U* = 25464.5; *p* < 0.001) where men scored significantly higher.

**TABLE 2:** Discrimination capacity between samples.

		TriPM	HSNS	LPFS_SR	NEF	DET	DISO/ANT	DIS	PSY	ANA
S1	Median	66	33	280	1.17	0.83	0.67	1.17	0.83	1
	Mean	68.91	32.33	283.52	1.23	0.92	0.8	1.22	0.86	1.06
	SD	22.65	5.73	68.21	0.71	0.59	0.66	0.66	0.62	0.61
S2	Median	47	28	203	0.67	0.5	0.33	0.5	0.33	1.17
	Mean	48.84	28.43	215.55	0.77	0.52	0.46	0.65	0.44	1.16
	SD	15.63	5.63	54.68	0.6	0.47	0.47	0.49	0.45	0.57
Mann–Whitney U		54922.5***	71161.0***	50962.5***	56863.5***	53899.5***	62880.0***	44723.5***	53551.0***	82267.5*

S1 and S2, Sample 1 and Sample 2; NEF, Negative Affectivity; DET, Detachment; DISO/ANT, Dissociality/Antagonism; DIS, Disinhibition; PSY, Psychoticism; ANA, Anankastia. *p < 0.05; ***p < 0.001.

### Convergent validity

The correlations between the scales in both samples are shown in Table 3. In Sample 1, the significant correlations ranged from very low *r* = 0.13 (Detachment–Dissociality/Antagonism) to high *r* = 0.92 (LPFS-SR total score–Identity^4^). In Sample 2, the significant correlations ranged from very low *r* = 0.09 (TriPM total score–HSNS total score) to high *r* = 0.92 (LPFS-SR total score–Identity). Interestingly, despite numerous intercorrelations, none of the variables in either sample correlated with the Anankastia domain. There was also no correlation between the Negative Affectivity domain–TriPM total score in any of the samples. Although the differences in correlation coefficients between the two samples were rather small, three exceptions were noted. The samples differed in the correlations Detachment–Dissociality/Antagonism; Detachment–TriPM total score; and TriPM total score–HSNS total score, while in all cases respondents in Sample 2 demonstrated more significant correlations.

**TABLE 3:** Correlations between scales in both samples.

		NEF	DET	DIS	DISO/ANT	PSY	ANA	LPFS-SR total	Identity	Self-direction	Empathy	Intimacy	TriPM total	HSNS total
NEF	S1	−												
	S2	−												
DET	S1	0.32***	−											
	S2	0.38***	−											
DIS	S1	0.56***	0.37***	−										
	S2	0.57***	0.40***	−										
DISO/ANT	S1	0.28***	0.13*	0.31***	−									
	S2	0.27***	0.44***	0.40***	−									
PSY	S1	0.46***	0.32***	0.58***	0.46***	−								
	S2	0.45***	0.45***	0.55***	0.50***	−								
ANA	S1	–0.01	0.02	–0.01	0.00	0.02	−							
	S2	0.03	–0.03	0.04	–0.08	–0.04	−							
LPFS-SR Total	S1	0.62***	0.49***	0.59***	0.34***	0.53***	0.03	−						
	S2	0.61***	0.56***	0.62***	0.47***	0.56***	0.03	−						
Identity	S1	0.64***	0.37***	0.59***	0.30***	0.52***	0.04	0.92***	−					
	S2	0.66***	0.49***	0.63***	0.40***	0.53***	0.03	0.93***	−					
Self-Direction	S1	0.55***	0.48***	0.54***	0.26***	0.42***	0.07	0.90***	0.78***	−				
	S2	0.52***	0.49***	0.60***	0.41***	0.48***	0.02	0.90***	0.81***	−				
Empathy	S1	0.46***	0.44***	0.49***	0.35***	0.49***	0.06	0.86***	0.71***	0.70***	−			
	S2	0.45***	0.48***	0.51***	0.43***	0.54***	–0.01	0.86***	0.71***	0.74***	−			
Intimacy	S1	0.53***	0.49***	0.48***	0.32***	0.48***	–0.05	0.87***	0.69***	0.68***	0.72***	−		
	S2	0.51***	0.55***	0.41***	0.45***	0.47***	0.03	0.88***	0.73***	0.69***	0.74***	−		
TriPM Total	S1	0.07	0.02	0.34***	0.57***	0.39***	0.06	0.26***	0.23***	0.20***	0.24***	0.27***	−	
	S2	–0.00	0.13**	0.26***	0.49***	0.32***	0.00	0.30***	0.24***	0.28***	0.33***	0.26***	−	
HSNS Total	S1	0.47***	0.30***	0.39***	0.28***	0.38***	–0.05	0.55***	0.56***	0.41***	0.48***	0.47***	0.09	−
	S2	0.55***	0.39***	0.44***	0.29***	0.38***	0.03	0.55***	0.54***	0.45***	0.47***	0.50***	0.09*	−

S1 and S2, Sample 1 and Sample 2; NEF, Negative Affectivity; DET, Detachment; DISO/ANT, Dissociality/Antagonism; DIS, Disinhibition; PSY, Psychoticism; ANA, Anankastia. *p < 0.05; **p < 0.01; ***p < 0.001.

### The addiction type specificity

Based on the available data, we determined four groups of patients according to the type of addiction within Sample 1. The first group consisted of patients with mixed addiction to alcohol and illicit drugs. The second group consisted of patients addicted only to alcohol, the third patients addicted only to illicit drugs, and the fourth gamblers and prescribed medication abusers. In the case of TriPM, groups differed by addiction type in all TriPM domains, i.e., Boldness (*p* = 0.019), Meanness (*p* = 0.031), and Disinhibition (*p* = 0.006). In relation to the HSNS, no difference was found between the groups with respect to the type of addiction (all *p*-values > 0.05). In the case of LPFS-SR, the difference was noticeable only in the Self-Direction domain (*p* = 0.036). The *post-hoc* test showed that only the third and fourth groups differ, i.e., the “only drugs” group (*M* = 77.79) and the “no alcohol/drugs” group (*M* = 64.13; *p* = 0.042). Finally, no difference was found in any of the six PID5BF+M domains by addiction type (all *p*-values > 0.05).

## Discussion

The current study primarily aimed to assess the capacity of the ICD-11 model for PDs to capture the features of narcissistic psychopathology in patients with addiction and the general population volunteers. We employed self-reported LPFS-SR and PID5BF+M as measurements of the general personality impairment and trait domains qualifiers, respectively. Moreover, we used TriPM and HSNS as measures of two types of pathological narcissism (i.e., grandiose, and vulnerable) in predicting the latent narcissistic features within the LPFS-SR and six domains of the PID5BF+M. In addition, our results contribute to the current state of knowledge on the clinical utility of the new model in terms of its ability to differentiate between clinical and non-clinical populations, gender, and to some extent, addicted patients depending on the type of addiction.

Despite the overlap between the DSM-5 AMPD and the ICD-11 model for PDs, a necessary first step from the perspective of personality trait qualifiers was the transposition of the original 220-item PID-5 into PID5BF+M, allowing the ICD-11 Anankastia qualifier to be evaluated. As the factor structure and internal consistency of the PID5BF+M published in earlier studies (40, 41) were confirmed for both of our samples, it was possible to consider this instrument as valid and reliable for our further analyses.

With regard the main objective of the study, our findings that almost 50% of LPFS-SR variability can be explained based on HSNS and TriPM confirm the fact that the core of the problem of pathological narcissism lies in a disturbed relationship with self and others (6) and thus underline the importance that the ICD-11 model for PDs places on the evaluation of personality functioning (56). On the other hand, related to the issue of SUDs, our results are somewhat contrary to the hypothesis of Salazar et al. (26) on the regulation of self-esteem by means of self-inducing a “narcissistic state,” according to which individuals take drugs to boost their inherent feeling of superiority over others. Since the HSNS score in Sample 1 proved to be the main predictor of LPFS-SR variability, it can be assumed that SUD patients in our study have rather low self-esteem associated with inferiority features of vulnerable narcissism. From the point of view of PID5BF+M, the expected associations with the Negative Affectivity and Dissociality/Antagonism domains were confirmed for both samples in relation to narcissistic psychopathology (23), although the latter domain was not confirmed as dominant. From the perspective of trait qualifiers, the relatively high capacity of the Disinhibition domain in capturing narcissistic pathology, which is usually associated with borderline PD rather than NPD, can be somewhat surprising. On the other hand, Clarkin et al. (57) point to the fact that low level borderlines are likely to have borderline PD with co-morbid narcissistic, paranoid, and antisocial PD or traits. In terms of anankastic traits, in contrast to previously postulated conclusions about perfectionistic overcompensation and rule-bound narcissistic dominance (58), no association between the Anankastia domain and both external measures of narcissistic psychopathology (i.e., HSNS and TriPM) was demonstrated in our study. Our results rather confirm earlier findings that NPD was present to a very low degree or absent in individuals with OCD (2). Although anankastic features can undisputable be found also in individuals with NPD, from the perspective of object relations theory, narcissistic disorders such as dissocial/antisocial PD, the syndrome of malignant narcissism, or NPD *per se* may be conceptualized along a continuum of rather extraverted pole of borderline personality organization spectrum, based on the degree of superego pathology, the intensity of projective defenses, and the presence of ego-syntonic aggression (59). In contrast, obsessive-compulsive PD is located within the introverted pole of neurotic personality organization (60). In this regard, our findings on the Anankastia domain can be interpreted that, although links between anankastic manifestations and manifestations of pathological narcissism may be observable at the symptom level, there is no direct connection between the two clinical phenomena in the context of deeper personality functioning. Other observed differences in magnitudes of statistical significance between the two samples (especially in relation to TriPM domains) can be interpreted in context of the greater sensitivity of the clinical sample in the detection of subtle psychopathological phenomena.

Considering the previously confirmed capacity of the original five domains of PID-5 to distinguish between clinical and non-clinical populations (61), it is not surprising that it was also confirmed in this study. Nevertheless, it is interesting that the Anankastia domain was the only one to show a lower statistical significance compared to the other PID5BF+M domains and scales. Similar problem was recently reported by Hemmati et al. (62). It can be considered that the orderliness, rigidity, and perfectionism facets may point to more desirable personality traits that can be found in individuals from the general population to a similar extent as in addiction patients. In other words, the Negative Affectivity, Detachment, Dissociality/Antagonism, Disinhibition, and Psychoticism domains, as well as the LPFS-SR, HSNS, and TriPM, may better address maladaptive variants of personality features that can be predicted to manifest more prominently in the clinical population.

From the perspective of gender differences, our results somewhat contradict the conclusions of the meta-analysis by Grijalva et al. (63), which showed that while men show more often than women manifestations of grandiose narcissism with signs of aggression and antagonism, in the case of vulnerable narcissism, gender differences were not demonstrated. However, the authors point out that these conclusions may be largely conditioned by the lack of studies verifying the manifestations of vulnerable narcissism in relation to gender. The predominance of men in manifestations of grandiose narcissism based on the TriPM was also evident in our study in both, the clinical and non-clinical sample (in the case of a general population sample in connection with the Dissociality/Antagonism domain). However, vulnerable narcissism based on the HSNS showed to be significant in both samples in women, in connection with the Negative Affectivity domain. In this regard, our results can be seen as further contribution to the empirical discussion on the issue of gender specificity of vulnerable narcissism in connection with five-factor models of personality. Considering Negative Affectivity as the negative pole of the Big Five trait of neuroticism (64), our findings are in line not only with Pincus et al. (65), who postulated the vulnerable narcissism as a negative affect-laden form of narcissism that is positively associated with the Big Five trait of neuroticism, but also with assumption about higher levels of neuroticism (i.e., less emotional stability) among women (66). Thus, it can be hypothesized that just as a higher level of Dissociality/Antagonism can increase the level of grandiose narcissism in men, so a higher level of Negative Affectivity can increase the level of vulnerable narcissism in women. However, this hypothesis requires further validation in both, clinical and non-clinical samples. In line with the conclusion of Green et al. (36) that gender may play a key factor that partly determines specific psychopathological constellations, our findings regarding the prevalence of vulnerable narcissistic features among women shed light on the specifics of addiction treatment in women. From a clinical perspective, the question remains whether unified regimen treatments based on discipline and order may not further increase the feeling of inferiority and thus dependence on authority, rather than promoting the growth of healthy female self-confidence and independence.

In terms of convergent validity, our results in general confirm the hypothesis of a strong common factor of PD severity, which might be scaled along a single latent continuum by measures specifically developed or adapted to capture the personality psychopathology within the ICD-11 model for PDs, i.e., PID5BF+M and LPFS-SR (67). From the perspective of the ICD-11 model, however, the Anankastia domain deserves special attention, while did not show any significant correlations with the other PID5BF+M domains or scales. Given that Anankastia in the ICD-11 model is intended to serve primarily as a qualifier of anankastic/obsessive-compulsive PD (23), which simultaneously exhibits the least impairment of personality functioning in relation to work, social relationships, and leisure activities when compared to other ICD-10 PDs (68), it can be hypothesized, that unlike the other ICD-11 qualifiers of personality traits, Anankastia is the only one that does not contain information related to the general personality structure adopted from the Five-Factor Model of personality (35). As such, it can contribute unique information about the patient’s maladaptive manifestations in relation to perfectionism, rigidity, and orderliness only if it is interpreted in the context of a separately assessed level of impairment in the overall personality functioning of the given individual (69). The absence of a significant correlation between the Negative Affectivity domain and the TriPM total score in both samples can be seen as expected given that the TriPM is a tool primarily intended for the evaluation of externalizing features of personality psychopathology (48), while Negative Affectivity affects symptomatic manifestations from the area of the internalizing spectrum (70). Differences in the magnitude of some correlations between Sample 1 and Sample 2 can be again attributed to the greater sensitivity of the clinical sample in the detection of subtle psychopathological phenomena. As all three aforementioned differences address correlations between opposing constructs (see Section “Results”), none of them appear to be clinically significant.

Finally, regarding addiction type specificity, our results pointing to differences between groups according to the type of addiction in TriPM domains can be interpreted from the point of view of the different degree of externalizing behavior in patients with SUD. This assumption is somewhat consistent with the conclusions of Few et al. (71) on the influence of externalizing behavior on variability in alcohol, drug use and antisocial behavior. Since the groups of alcohol-dependent and polysubstance-dependent patients were the most represented, our results in relation to TriPM underline the importance of clinical evaluation of externalizing behavior within a standard addiction setting. Adopting an object relations model perspective of narcissism and psychopathy (72), a thorough examination of the degrees of deficits in moral functioning appears to be a beneficial procedure for capturing the level of superego pathology that may have a significant impact on treatment outcome and relapse prevention. It can be hypothesized that as the degree of severity of superego dysfunction increases, the patient’s willingness to adhere to abstinence and recommended treatment procedures will decrease if there is no immediate external personal gain. This assumption could be confirmed also in the case of illicit drug users, although it requires further verification in better balanced samples of addicted patients according to the substance used. Regarding the difference between groups noted in relation to LPFS-SR, because the group without alcohol/drugs addiction consisted largely of gamblers, the difference between the pure drug users’ group and no alcohol/drugs addiction group in LPFS-SR Self-Direction domain may be attributed to individual differences in features of impulsivity and compulsivity (73) rather than more general differences in the ability to set and adhere to short-term and long-term goals. However, the possibility of a statistical artifact cannot be excluded due to the low number of pure drug users in our clinical sample (*n* = 14).

The results of our study contribute to the current state of knowledge in several ways. First, we confirmed the capacity of the LPFS-SR and most of the PID5BF+M domains in detecting features of vulnerable/hypersensitive and grandiose narcissism; second we have confirmed the ability of the ICD-11 model for PDs to differentiate between clinical and non-clinical populations based on self-reported LPFS-SR and PID5BF+M; third, our findings support the gender specificity toward the two prototypes of pathological narcissism; fourth, we highlighted the interconnections between both crucial parts of the ICD-11 model, i.e., the overall level of impairment in personality functioning and PD trait qualifiers with exception for Anankastia domain; and finally fifth, our findings illuminate some personality specifics between the subgroups of addicted patients in relation to the type of addiction. However, our findings should be considered in light of certain limitations that may inspire future research. We consider the method of data collection itself to be a significant limitation of the study. The battery of questionnaires was very comprehensive and made great demands on the participants’ time and ability to concentrate. In this regard, the so-called non-response bias could have occurred in some cases, which according to Sedgwick (74) is the most common limit in questionnaire surveys. Respondents may miss the question, or miss it on purpose, and the reasons may be socio-cultural, or in behavior and attitudes. Although we subjected the data to a thorough cleaning procedure, it can’t be ruled out that a different order of the questionnaires or their distribution into two shorter sets could have a positive effect on their quality, especially regarding the number of missing items in some scales. Another limitation may be our focus on addicted patients from high-threshold services. This choice was deliberate regarding the high risk of dropout and acute intoxication in low-threshold clientele. On the other hand, we are aware that by excluding this large group of patients with addiction, there was an impoverishment of data on psychopathological phenomena that can have a significant influence in relation to narcissism. Clinical practice confirms that low-threshold clientele shows a higher degree of personality pathology and criminal behavior. Thus, future research could significantly enrich our findings by including these patients. From the perspective of expanding the clinical implications of our findings into addiction settings, future research should focus on more detailed analyses of differences in the type of addiction with an emphasis on a more balanced groups of addicted patients. Finally, reliance on self-report data on pathological narcissism and NPD may be a limitation. Both grandiosity and vulnerability can hinder accurate self-assessment and affect the accuracy of assessment of individual personality pathology. From this perspective, supplementing the assessment with, for example, the Structured Interview of Personality Organization-Revised (STIPO-R) (75), including an index for the assessment of pathological narcissism, seems to be a suitable solution.

## Data availability statement

The raw data supporting the conclusions of this article will be made available by the authors, without undue reservation.

## Ethics statement

The studies involving human participants were reviewed and approved by Ethics Committee of the General University Hospital in Prague Na Bojišti 1, 128 08 Prague 2. The patients/participants provided their written informed consent to participate in this study.

## Author contributions

KDR drafted the manuscript, supported the data analyses, and was responsible for its final version. LS was responsible for data collection. TSZ wrote parts of the introduction and discussion and critically revised the first draft. All authors contributed to the article and approved the submitted version.

## Footnotes

1 We use the term “SUD” when referring specifically to substance addictions, while the term “addiction” is used when pointing to addictions in general.

2 The Anankastia domain is only part of the ICD-11 model whereas the Psychoticism domain is only part of the AMPD.

3 Total higher than 100% due to polyvalent addiction.

4 This correlation was verified to compare which of the LPFS-SR subscales better represents its total score. In this sense, it can be understood as a certain way of assessing the internal consistency of the LPFS-SR.
